# Grammar-based distance in progressive multiple sequence alignment

**DOI:** 10.1186/1471-2105-9-306

**Published:** 2008-07-10

**Authors:** David J Russell, Hasan H Otu, Khalid Sayood

**Affiliations:** 1 Department of Electrical Engineering, University of Nebraska-Lincoln, 209N WSEC, Lincoln, NE, 68588-0511, USA; 2 New England Baptist Bone and Joint Institute, Beth Israel Deaconess Medical Center Genomics Center, Harvard Medical School, Boston, MA 02215, USA

## Abstract

**Background:**

We propose a multiple sequence alignment (MSA) algorithm and compare the alignment-quality and execution-time of the proposed algorithm with that of existing algorithms. The proposed progressive alignment algorithm uses a grammar-based distance metric to determine the order in which biological sequences are to be pairwise aligned. The progressive alignment occurs via pairwise aligning new sequences with an ensemble of the sequences previously aligned.

**Results:**

The performance of the proposed algorithm is validated via comparison to popular progressive multiple alignment approaches, ClustalW and T-Coffee, and to the more recently developed algorithms MAFFT, MUSCLE, Kalign, and PSAlign using the BAliBASE 3.0 database of amino acid alignment files and a set of longer sequences generated by Rose software. The proposed algorithm has successfully built multiple alignments comparable to other programs with significant improvements in running time. The results are especially striking for large datasets.

**Conclusion:**

We introduce a computationally efficient progressive alignment algorithm using a grammar based sequence distance particularly useful in aligning large datasets.

## Background

Generation of meaningful multiple sequence alignments (MSAs) of biological sequences is a well-studied NP-complete problem, which has significant implications for a wide spectrum of applications [1,2]. In general, the challenge is aligning *N *sequences of varying lengths by inserting gaps in the sequences so that in the end all sequences have the same length. Of particular interest to computational biology are DNA/RNA sequences and amino acid sequences, which are comprised of nucleotide and amino acid residues, respectively.

MSAs are generally used in studying phylogeny of organisms, structure prediction, and identifying segments of interest among many other applications in computational biology [3].

Given a scoring scheme to evaluate the fitness of an MSA, calculating the best MSA is an NP-complete problem [1]. Variances in scoring schemes, need for expert-hand analysis in most applications, and many-to-one mapping governing elements-to-functionality (codon mapping and function) make MSA a more challenging problem when considered from a biological context as well [4].

Generally, three approaches are used to automate the generation of MSAs. The first offers a brute-force method of multidimensional dynamic programming [5], which may find a good alignment but is generally computationally expensive and, therefore, unusable beyond a small *N*. Another method uses a probabilistic approach where Hidden Markov Models (HMMs) are approximated from unaligned sequences. The final method, progressive alignment, is possibly the most commonly used approach when obtaining MSAs [6].

A progressive alignment algorithm begins with an optimal alignment of two of the *N *sequences. Then, each of the remaining *N *sequences are aligned to the current MSA, either via a consensus sequence or one of the sequences already in the MSA. Variations on the progressive alignment method include PRALINE [7], ProbCons [8], MAFFT [9,10], MUSCLE [11,12], T-Coffee [13], Kalign [14], PSalign [15], and the most commonly used CLUSTALW [16]. In most cases, the algorithms attempt to generate accurate alignments while minimizing computational time or space. Advances in DNA sequencing technology with next generation sequencers such as ABI's SOLID and Roche's GC FLX provide vast amount of data in need of multiple alignment. In case of large sequencing projects, high number of fragments that lead to longer contigs to be combined are generated with much less time and money [17]. In addition, as more organisms' genomes are sequenced, approaches that require MSA of the same gene in different organisms now find a more populated data set. In both cases computational time in MSA is becoming an important issue that needs to be addressed.

This work presents GramAlign, a progressive alignment method with improvements in computational time. In particular, the natural grammar inherent in biological sequences is estimated to determine the order in which sequences are progressively merged into the ongoing MSA. The following sections describe the algorithm and present initial results as compared with other alignment algorithms.

## Methods

**Figure 1:**
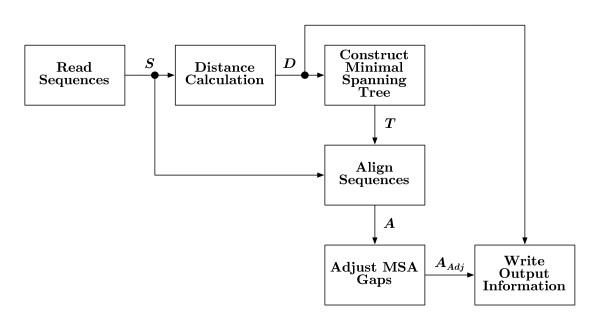
**Algorithm overview**. The algorithm operates on a set of sequences *S *originally read in FASTA format. After a grammar-based distance matrix *D *is estimated, a minimal spanning tree *T *is constructed. The tree is used as a map for determining the order in which the sequence set is progressively aligned in *A*. Gaps in the alignment are grouped together using a sliding window resulting in *A*_*Adj*_. Several outputs are available, including the distance matrix and various sequence alignment formats.

A general overview of the GramAlign algorithm is depicted in Figure 1. The set of sequences to be aligned, *S*, are regarded as input to the algorithm with *S *= {*s*_1_,...,*s*_*N*_}, where *s*_*i *_is the *i*^*th *^sequence and *i *∈ {1,...,*N*}.

### Distance Estimation

The first step in the procedure involves the formation of an estimate of the distance between each sequence *s*_*m *_and all other sequences *s*_*n *_∀ *n *≠ *m*. The distance used in GramAlign is based on the natural grammar inherent to all information-containing sequences. Unfortunately, the complete grammar for biological sequences is unknown, and so cannot be used when comparing sequences. However, we do know that biological sequences have structures which correspond to functions. This in turn implies that biological sequences which correspond to proteins with similar functions will have similarities in their structure. Therefore, we use a grammar based on Lempel-Ziv (LZ) compression [18,19] used in [20] for phylogeny reconstruction. This measure uses the fact that sequences with similar biological properties share commonalities in their sequence structure. It is also known that biological sequences contain repeats, especially in the regulatory regions [21]. When comparing sequences with functional similarity, non-uniform distribution of repeats among the sequences poses a problem to assess sequence similarity. As shown below, the proposed distance naturally handles such cases, which are difficult to be accounted for by alignment or sequence edit based measures.

**Figure 2:**
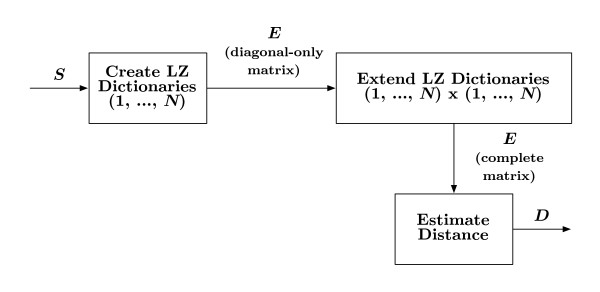
**Distance calculation**. An *N *× *N *grammar-based distance matrix *D *is estimated from the set of *N *input sequences *S*. The first step in generating *D *is to approximate the original number of elements in each sequence's dictionary based on an LZ complexity. Each dictionary is extended using all other sequences resulting in new numbers of elements. The grammar-based distance between sequences *m *and *n *is determined by considering the amount by which dictionaries change.

An overview of the grammar-based distance calculation is shown in Figure 2 where a dictionary of grammar rules for each sequence is calculated. Initially, the dictionary $G_{m}^{1}$ = ∅ is empty, a fragment *f*^1 ^= *s*_*m*_(1) is set to the first residue of the corresponding sequence, and only the first element *s*_*m*_(1) is visible to the algorithm. At the *k*th iteration of the procedure, the *k*th residue is appended to the *k *- 1 fragment and the visible sequence is checked. If *f*^*k *^∉ *s*_*m*_(1,...,*k *- 1) then *f*^*k *^is considered a new rule, and so added to the dictionary $G_{m}^{k}=G_{m}^{k-1}\cup \{f^{k}\}$, and the fragment is reset, *f*^*k *^= ∅. However, if *f*^*k *^∈ *s*_*m *_(1,...,*k *- 1), then the current dictionary contains enough rules to produce the current fragment, i.e., $G_{m}^{k}=G_{m}^{k-1}$. In either case, the iteration completes by appending the *k*th residue to the visible sequence. This procedure continues until the visible sequence is equal to the entire sequence, at which time the size of the dictionary is recorded along the diagonal of the grammar elements matrix, *E*_*m, m *_= |*G*_*m*_|. As will be shown, calculating the distance between sequences requires only the number of entries in the dictionary.

In the next step shown in Figure 2, each sequence is compared with all other sequences. In particular, consider the process of comparing sequences *m *and *n*. Initially, the dictionary $G_{m,n}^{1}$ = *G*_*m *_is set to that of sequence *m*, a fragment *f*^1 ^= *s*_*n*_(1) is set to the first residue of the *n*th sequence, and the visible sequence is all of *s*_*m*_. The algorithm operates as described previously, resulting in a new dictionary size *E*_*m, n *_= |*G*_*m,n*_|. When complete, more grammatically-similar sequences will have a new dictionary size with fewer entries as compared to sequences that are less grammatically-similar. Therefore, the size of the new dictionary *E*_*m*,*n *_will be close to the size of the original dictionary *E*_*m,m*_.

In the final step, the distance between the sequences is estimated using the dictionary sizes. Five different distance measures were suggested in [20]. This work used the distance measure

(1) $$d_{m,n}=\frac{E_{m,n}-E_{m,m}+E_{n,m}-E_{n,n}}{\frac{E_{m,n}+E_{n,m}}{2}},$$

where *m*, *n *∈ {1,...,*N*} are indices of two sequences being compared. This particular metric accounts for differences in sequence lengths, and normalizes accordingly. Thus, the final distance matrix *D *is composed of grammar-based distance entries given by (1). Smaller entries in *D *indicate a stronger similarity, at least in terms of the LZ-based grammar estimate. Intuitively, sequences with a similar grammar should be pairwise aligned with each other in order for progressive combining into an MSA.

To further improve the execution time, *D *is only partially calculated as follows. An initial sequence is selected and compared with all other sequences. The resulting distances are split evenly into two groups based on *d*, one containing the smallest distances, and the other containing the largest distances. The process is repeated recursively on each group until the number of sequences in a group is two. The benefit is that only *N *log(*N*) distances need to be calculated. The validity of only calculating these sets of distances stems from the transitivity of the LZ grammars being inferred. That is, if the grammar-based distances *d*_*i, j *_and *d*_*j, k *_are small, it is likely that *d*_*i, k *_is also small. By recursively dividing groups of extreme distances, only those distances which would likely be used in the spanning-tree creation process will actually be calculated.

#### Sequence Alphabet

**Figure 3:**
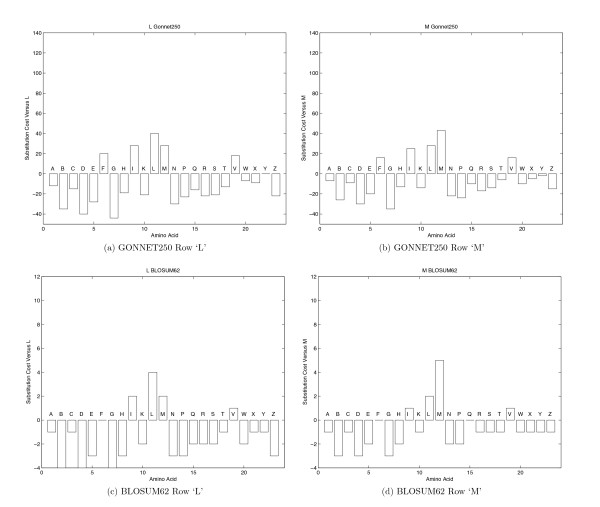
**Substitution scores for amino acid 'L' and 'M'**. Bar graphs of the substitution scores for amino acid 'L' and 'M' as taken from the Gonnet250 and BLOSUM62 substitution matrices. The scores are shown based on an alphabetical ordering of amino acid letters from the leftmost 'A' to rightmost 'Z'.

The distance between sequences *m *and *n *as determined by (1) is based on how many additional rules need to be added to each grammar in order to generate both *s*_*m *_and *s*_*n*_. Because the real grammars are unknown, *G*_*m *_and *G*_*n *_are approximated by scanning the only observations available (i.e., *s*_*m *_and *s*_*n*_). The grammar approximation improves as the length of the observed sequences increases. And so, the distance calculations are a function of sequence lengths, becoming more accurate as the sequences increase in length. In practice, this calculation works well for DNA sequences, even of shorter lengths, because the approximated grammar of a DNA sequence can only contain rules involving words composed of combinations of elements from the alphabet {'A','C','G','T'}. This small alphabet allows for a rapid generation of a reasonable grammar since there are a relatively small number of permutations of letters. From a grammar perspective, amino acid sequences are generally much more difficult to process correctly using (1). The reason being the alphabet contains 23 letters, where each element is not equally different from all other elements. Due to the relatively large alphabet size, much longer sequences are necessary to generate a reasonable grammar approximation. Thus, the accuracy of distances calculated for sets of short amino acid sequences are diminished. Additionally, consider the substitution scores of 'L' and 'M' as taken from the GONNET250 and BLOSUM62 substitution matrices in Figure 3. Notice in (a) and (c), that 'L' receives a relatively high positive value when aligned with any of {'I','L','M','V'}. Similarly, in (b) and (d), 'M' receives a relatively high positive value when aligned with any of the same set. Additionally, both 'L' and 'M' generally receive high negative values when compared to letters other than {'I','L','M','V'}. When taking this type of scoring into account, the elements 'L' and 'M' could be considered the same letter in a grammatical sense.

Thus, GramAlign offers the option to use a "Merged Amino Acid Alphabet" when calculating the distance matrix. The merged alphabet contains 11 elements corresponding to the 23 amino acid letters grouped into the sets {'A','S','T','X'}, {'B','D','N'}, {'C'}, {'E','K','Q','R','Z'}, {'F'}, {'G'}, {'H'}, {'I','L','M','V'}, {'P'}, {'W'}, and {'Y'}. These groupings were determined by considering all 23 rows of the BLOSUM45, BLOSUM62, BLOSUM80 and GONNET250 substitution matrices, and only grouping elements that had a strong similarity across the entire row in all four matrices. The merged alphabet has the benefit of containing fewer elements allowing for more accurate distance estimates based upon shorter observed sequences. Also, the resultant merged-alphabet substitution matrices are more consistent in that a merged-letter score is high only when compared to itself. In practice, the average alignment scores increased when aligning the same data sets using the merged alphabet within the distance calculation, as compared to using the actual alphabet (results not shown). In either case, once the distances have been calculated, a tree based on these distances is used to determine which sequences should be pairwise aligned.

### Tree Construction

The next step in the algorithm consists of constructing a minimal spanning tree *T *based on the distance matrix *D*. In particular, consider a completely connected graph of *N *vertices and $\frac{N(N-1)}{2}$ edges, where the weight of an edge between vertices *i *and *j *is given by the (*i, j*)th element of the distance matrix, *D*_*i, j*_. This work uses Prim's Algorithm [22] to determine a minimal spanning tree *T *which may be used as a guide in determining the order for progressively aligning the set of sequences *S*.

### Align Sequences

**Figure 4:**
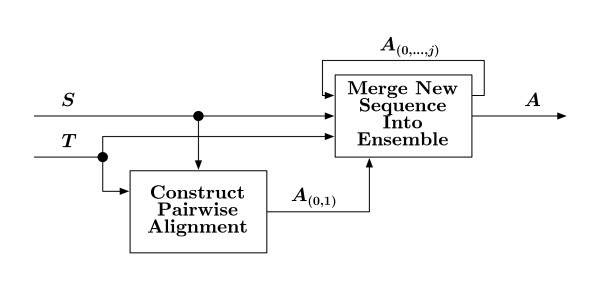
**Align sequences**. From the spanning tree *T *and the set of sequences *S*, a progressive alignment is generated and stored in an ensemble. When no more sequences remain, the final alignment *A *is available for post-processing gap adjustments.

The minimal spanning tree *T *along with the set of sequences *S*, are processed by the "Align Sequences" block in Figure 1. This block is presented in more detail in Figure 4. The first two sequences from *S *to be aligned are given by *T *as the root sequence of *T *and the nearest sequence in terms of the LZ grammar distance. At the conclusion of the pairwise alignment process, the resulting alignment is stored in an ensemble of sequences.

In the following we describe the pairwise alignment procedure, the scoring system and the method for progressive alignment.

#### Dynamic Programming

At the core of most progressive MSA algorithms is some method for performing pairwise alignments between two sequences. This work uses a version of the [23] dynamic programming algorithm with affine gap scores as discussed in [2] to generate each pairwise alignment.

#### Scoring System

A significant ambiguity regarding the dynamic programming procedure is the scoring function used when comparing two elements, or when comparing an element with a gap.

Typically, the pairwise scoring function *c*() is simply a matrix of values, where each column and row represent one element in the alphabet. In this way, each cell of the matrix corresponds to some measure representing the likelihood that two sequence elements should be aligned with each other. The most well-known amino acid scoring matrices are the Percent Accepted Mutation (PAM) [24], BLOck SUbstitution Matrix (BLOSUM) [25] and GONNET [26]. GramAlign defaults to the GONNET250 substitution matrix for the scoring function *c*(), as other progressive alignment algorithms generally use it as the default choice (e.g., [14] and [16]).

Determining the best gap-open and gap-extension penalties is a challenging problem, made more difficult by introducing two different penalties to account for the beginning and ending tail gaps of alignments. The default gap penalties used by GramAlign have been adjusted to perform well based on the alignment sets presented in the results section.

#### Progressive Alignment

The ensemble is implemented as a doubly-linked list, where each node of the list represents a single column of the alignment. Each node of the ensemble contains an array of letters corresponding to the respective column alignment, a tally of gaps in the column, a weighted combination of substitution scores, and two gap penalties. Once the initial ensemble *A*_(0,1) _is constructed between the first two entries in *T*, the remaining sequences need to be added to the ensemble in the order defined by *T*. This is accomplished by checking *T *for the next sequence not already in the ensemble, call it sequence *s*_*j *_where *j *corresponds to the order in which the sequence was added to *T*; that is, *j *is the priority of the sequence. To progressively add *s*_*j *_to the alignment, a pairwise alignment between the ensemble *A*_(0,...,*j*-1) _and *s*_*j *_is created via the afore mentioned dynamic programming algorithm. While the algorithm used is a pairwise alignment algorithm the distance calculated at each step of the pairwise alignment is an average of the distances between the particular position being aligned in the new sequence and the corresponding amino acides or bases in the ensemble at that node. The new pairwise alignment is merged into the ongoing ensemble based on the trace-back. The process continues until all sequences have been added to the ensemble of sequences. When sequence *s*_*j *_is added to the current ensemble *A*_(0,...,*j*-1)_, each node is updated to reflect the new column element.

### Gap Adjustment

**Figure 5:**
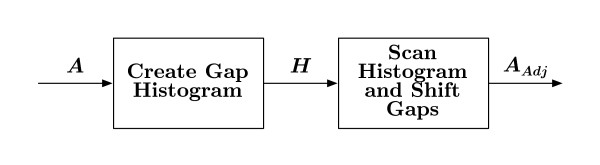
**Adjust gaps**. Gaps in the complete MSA ensemble *A *are grouped together via a sliding window. After the histogram *H *of gaps-per-column is generated, an equidistant column-window is shifted across the alignment, moving one column per interval. If the center column contains more gaps than some parameter threshold, the columns within the window are scanned for possible gaps that may be shifted into the center column. The resulting adjusted ensemble *A*_*Adj *_is presented as the final alignment.

Once all *N *sequences have been progressively aligned, the final post-processing block in Figure 1, "Adjust MSA Gaps", is used to cluster gaps together. The adjustment is further detailed in Figure 5, where the ensemble *A *is scanned so a histogram *H *of gaps-per-column is generated.

The histogram *H *is scanned using an equidistant, user-adjustable sliding window about each column. For each column, when the number of gaps is greater than a user-adjustable threshold percentage of gaps-per-column, the following steps are taken. For each row in the column under consideration:

1. If the current row has a gap, move to the next row;

2. Otherwise, scan the current row of the neighboring columns within the window, beginning with the nearest columns and work outward;

3. If a neighboring column has a gap in the current row and the neighboring column has fewer total gaps than the center column, shift the gap from the neighboring column into the column under consideration.

As an illustration, consider a portion of the ensemble

$$A:\{\begin{array}{lllll} \ldots x_{1,i-2} & x_{1,i-1} & {-}_{1,i} & x_{1,i+1} & x_{1,i+2}\ldots \\ \ldots x_{2,i-2} & {-}_{2,i-1} & {-}_{2,i} & {-}_{2,i+1} & x_{2,i+2}\ldots \\ \ldots x_{3,i-2} & {-}_{3,i-1} & {-}_{3,i} & {-}_{3,i+1} & x_{3,i+2}\ldots \\ \ldots {-}_{4,i-2} & x_{4,i-1} & x_{4,i} & {-}_{4,i+1} & x_{4,i+2}\ldots \\ \ldots x_{5,i-2} & x_{5,i-1} & x_{5,i} & {-}_{5,i+1} & x_{5,i+2}\ldots \end{array}$$

where *x*_*m, n *_represents any element other than a gap in column *n *of sequence *m*, and -_*m, n *_represents a gap in column *n *of sequence *m*. And so, the gap histogram for this section of ensemble is *H *= {...,1, 2, 3, 4, 0,...}. Assuming the gap threshold is 0.4, then only columns with more than two gaps will be considered for adjustment. In the example, *H *is scanned until column *i *is identified as having three gaps. Following the procedure, each row in column *i *is checked until a non-gap entry is found. In the example, the first non-gap entry *x*_4, *i *_is in row four. Assuming the gap window is 2, elements in the fourth row of the neighboring columns are checked for gap entries. In particular, column (*i *+ 1) is checked first, with a gap entry -_4, *i*+1_. However, no shift occurs because a quick check of *H *shows that column (*i *+ 1) has more gaps than column *i*. Continuing the scan, columns (*i *- 1) and (*i *+ 2) are checked before another gap is found in column (*i *- 2). In this case, *H *indicates column (*i *- 2) has fewer gaps compared to column *i*, and so a blind shift of entries between (*i *- 2) and *i *occurs, resulting in the ensemble

$$A:\{\begin{array}{lllll} \ldots x_{1,i-2} & x_{1,i-1} & {-}_{1,i} & x_{1,i+1} & x_{1,i+2}\ldots \\ \ldots x_{2,i-2} & {-}_{2,i-1} & {-}_{2,i} & {-}_{2,i+1} & x_{2,i+2}\ldots \\ \ldots x_{3,i-2} & {-}_{3,i-1} & {-}_{3,i} & {-}_{3,i+1} & x_{3,i+2}\ldots \\ \ldots x_{4,i-2} & x_{4,i} & {-}_{4,i-2} & {-}_{4,i+1} & x_{4,i+2}\ldots \\ \ldots x_{5,i-2} & x_{5,i-1} & x_{5,i} & {-}_{5,i+1} & x_{5,i+2}\ldots \end{array}$$

where original indices are kept to depict which entries are shifted into which locations.

The result is a blind movement of sparse gaps into dense regions of gaps. Numeric simulations have shown this post-processing stage does not affect alignment scoring based upon the method used in the Results and Discussion section (results not shown). And so, the user-defined parameters are set to a threshold of 1.0 and a window of 0 columns by default thereby disabling the gap adjustment block. Should it be known there are conserved regions of gaps, the user may decide to enable this process to encourage gap grouping.

### Algorithm Complexity

The algorithm complexity of GramAlign may be broken into five pieces, beginning with the generation of each sequence grammar dictionary, *G*_*i *_for *i *∈ {1,...,*N*}, where *N *is the number of sequences. Suppose the average sequence length is *L*, then each *G*_*i *_results in complexity O(*L*), so all dictionaries are generated with complexity O(*LN*). Next, the distance matrix *D *is formed by recursively extending a grammar by all other sequences within it's neighborhood, each of which results in complexity O(*L*), then splitting the neighborhood into two halves, resulting in a complexity O(*LN *log(*N*)). The spanning tree *T *is constructed by searching over *D *with a complexity of O(*N*^2^). The tree is used as a map in determining the order in which to perform *N *- 1 pairwise alignments, each requiring a complexity of O(*L*^2 ^+ *L*). Thus, the progressive alignment process takes O(*L*^2^*N*). The alignment ensemble is scanned and has gaps shifted in O(*LN*) time. Thus, the entire time complexity for GramAlign is O(*LN *+ *LN *log(*N*) + *N*^2 ^+ *L*^2^*N *+ *LN*), which simplifies to O(*N*^2 ^+ *L*^2^*N*).

## Results and Discussion

In this section, example alignments are used to study the possible advantages of GramAlign. All results were generated by compiling and executing the respective MSA programs on the same computer; specifically, an Apple iBook with a PowerPC G4 operating at 1.2 GHz with 1.25 Gb system memory and 512 Kb L2 cache. Two sets of experiments were conducted. The first set of experiments were conducted using the unaligned FASTA files from the BAliBASE 3.0 [27] data-set, a well-accepted benchmark database containing example amino acid sequences. The resulting aligned FASTA files from each algorithm were scored using bali score, a program provided with the BAliBASE distribution that generates a Sum-of-Pairs (SP) score and a Total-Column (TC) score based on predetermined reference alignments. The size of the sequences in the BAliBASE distribution are relatively small and, therefore, not very useful in demonstrating the advantages to be obtained using a fast algorithm. The second set of experiments were conducted using sequences generated by Rose version 1.3 to demonstrate algorithms' capabilities on large data sets containing either long or numerous sequences. Rose is a software tool that implements a probabilistic model of sequence evolution, so that a user is able to generate families of related sequences from a common ancestor sequence via insertion, deletion and substitution [28]. Rose allows for many parameter adjustments including rate of mutation, desired average final sequence length and number of desired sequences. The tool outputs the unaligned sequences, as well as the real alignment based on how mutations occur, and an evolutionary tree. The set of sequences generated by Rose were based on the default seed file provided with the Rose software distribution, where the seed file is the method used to input parameters to Rose.

Note the use of simulated data here is to demonstrate the speed advantage of GramAlign, while maintaining a similar qualitative score. The default values were used to generate the data and the algorithms were not tuned to the data. The use of simulated data may actually provide a biased advantage in quality score to any given alignment program, depending on how the simulated data is generated. A wider breadth of simulated data, such as was done in [29], would provide a better assessment of overall alignment quality.

### BAliBASE Experiments

Alignment files in the BAliBASE database are separated into five categories (RV1x through RV50), each exhibiting different classes of alignment issues (e.g., one sequence might be significantly longer than the other sequences in a file). The first class is further divided into two subcategories labeled RV11 and RV12. The results presented in Table 1 and Table 2 respectively detail the average SP and TC scores over each category as aligned by GramAlign version 1.14 (see Additional file 1), ClustalW version 1.83, T-Coffee version 4.45, PSAlign using ProbCons as the tree generation (no version given, archive created on 3/2/2006), Kalign version 1.04, MAFFT version 5.861, and MUSCLE version 3.6. Additionally, a fast version was tested for ClustalW, MAFFT, MUSCLE and MAFFT version 6.240. In particular, the command line options used were clustalw -quicktree, mafft --retree 1, muscle -maxiters 1 -diags -sv -distance1 kbit20_3 and mafft --retree 1 --parttree --partsize 50 to incorporate high-speed progressive options. In all cases the default parameters were used for each program. In general, there are no significant differences in the performance of GramAlign and other algorithms as far as the SP and TC scores are concerned. As may be seen, GramAlign provides similar alignments in terms of the quality determined via the scoring method used.

**Table 1:** Average SP scores on BAliBASE.

Algorithm	RV11	RV12	RV20	RV30	RV40	RV50
MUSCLE (fast)	0.4904	0.8303	0.8359	0.7076	0.6904	0.6823
MAFFT (fast)	0.4801	0.8161	0.8404	0.7345	0.7187	0.7089
MAFFT v6 (parttree, n = 50)	0.4790	**0.8066**	**0.8096**	**0.6801**	**0.6610**	0.6985
MAFFT	0.4914	0.8258	0.8459	0.7437	0.7347	0.7253
GramAlign	0.5089	0.8328	0.8270	0.6855	0.7239	0.6903
Kalign	0.5029	0.8504	0.8410	0.7389	0.7259	0.7299
ClustalW (fast)	**0.4748**	0.8367	0.8258	0.6843	0.6705	0.6715
MUSCLE	0.5578	0.8583	0.8548	0.7492	0.7623	0.7384
ClustalW	0.4908	0.8197	0.8219	0.6841	0.6950	**0.6698**
PSAlign	**0.5924**	**0.8804**	**0.8720**	0.7554	**0.7937**	**0.7739**
T-Coffee	0.5181	0.8650	0.8660	**0.7588**	0.7452	0.7715

Average SP score for each algorithm for each category offered by the BAliBASE test suite. The bold entries indicate the lowest and highest scores.

**Table 2:** Average TC scores on BAliBASE.

Algorithm	RV11	RV12	RV20	RV30	RV40	RV50
MUSCLE (fast)	0.2421	0.6349	**0.2599**	**0.2457**	**0.2614**	0.2719
MAFFT (fast)	0.2354	**0.6209**	0.3094	0.2910	0.3108	0.3087
MAFFT v6 (parttree, n = 50)	0.2461	0.6320	0.2978	0.2987	0.3104	0.3435
MAFFT	0.2532	0.6256	0.3168	0.3158	0.3073	0.3303
GramAlign	0.2993	0.6701	0.2917	0.2503	0.3292	0.3006
Kalign	0.2538	0.6749	0.2765	0.2955	0.3253	0.3223
ClustalW (fast)	**0.2317**	0.6651	0.2680	0.2513	0.2808	0.2752
MUSCLE	0.3217	0.6961	0.3077	0.3087	0.3484	0.3397
ClustalW	0.2395	0.6417	0.2602	0.2478	0.3024	**0.2658**
PSAlign	**0.3503**	**0.7384**	**0.3517**	0.2992	**0.3951**	0.3816
T-Coffee	0.2716	0.6986	0.3257	**0.3637**	0.3659	**0.3974**

Average TC score for each algorithm for each category offered by the BAliBASE test suite. The bold entries indicate the lowest and highest scores.

Presented in Table 3 are the execution times necessary to generate the entire data presented in Table 1 and Table 2. GramAlign finishes in approximately 0.4% of the time needed by PSAlign, which generated the highest scoring alignments in five out of the six BAliBASE categories as far as SP scores are concerned. PSAlign's average SP and TC score on the other hand were 9.4 and 17.5% better than GramAlign's scores, which was approximately 223 times faster. Out of the four approaches MAFFT, MAFFT v6, MAFFT (fast), MUSCLE (fast), which were 17.1, 49.9, 54.0, and 55.7% faster than GramAlign, respectively, only MAFFT had a 2% better average SP score than GramAlign. All other average SP and TC scores were equivalent or worse than that of GramAlign. Further, the GramAlign alignments scored equal-to or greater-than 56.9, 59.6, 60.8, and 71.1% of the trials based on TC score, compared to MAFFT, MAFFT v6, MAFFT (fast), and MUSCLE (fast) (results not shown). GramAlign finishes in 33% of the time required by ClustalW using -quicktree, and only 8% needed by ClustalW, possibly the most widely used MSA program.

**Table 3:** Execution time.

Algorithm	Execution Time (sec)
MUSCLE (fast)	301
MAFFT (fast)	313
MAFFT v6 (parttree, n = 50)	341
MAFFT	564
GramAlign	680
Kalign	1,329
ClustalW (fast)	2,071
MUSCLE	6,129
ClustalW	8,720
PSAlign	152,168
T-Coffee	403,815

Execution time necessary to align all trials in the BAliBASE test suite.

### Experiments with Large Data Sets

#### Long Sequence Experiments

In order to compare the performance of MSA algorithms on long data sets, two sets of seven FASTA files each containing ten sequences were generated using Rose version 1.3. The first set of seven FASTA files contains protein sequences and the second set contains DNA sequences. In both sets, the first file contains sequences with an average length of 5,000 residues, with each file increasing the average sequence length by 5,000 residues. Thus, the seventh file contains ten sequences with an average sequence length of 35,000 residues.

**Figure 6:**
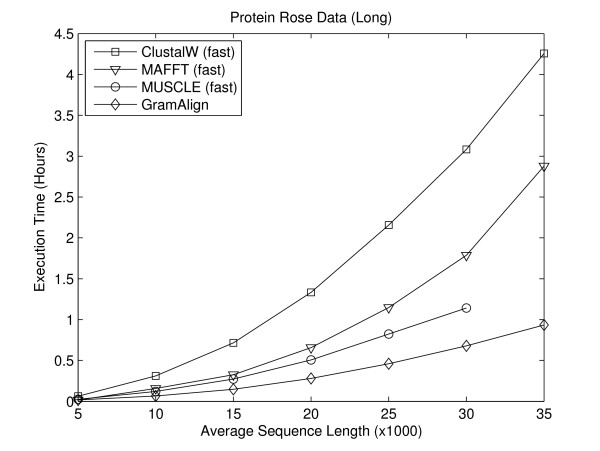
**Rose protein long**. Result of executing the fastest algorithms on the Rose-generated long protein sequence sets.

**Figure 7:**
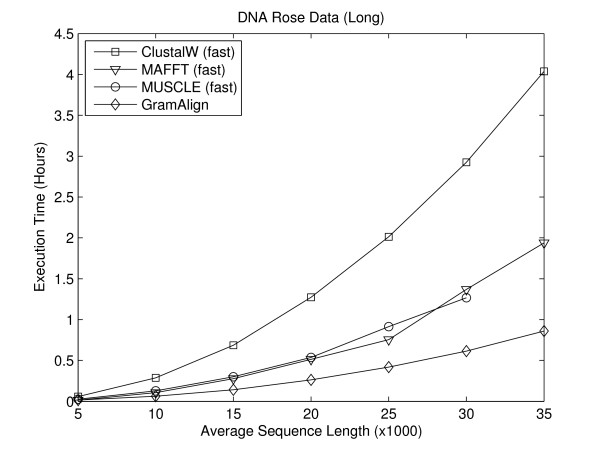
**Rose DNA long**. Result of executing the fastest algorithms on the Rose-generated long DNA sequence sets.

Figures 6 and 7 depict the execution time required for the fastest algorithms to align the seven large protein and DNA sequence sets, respectively. As the average length of sequences increases, the difference in time required by GramAlign compared to the other algorithms also increases. In particular, at an average sequence length of 35,000 residues GramAlign completes the alignments in 3,363 and 3,092 seconds, while the nearest algorithm (MAFFT in fast-mode) requires 10,362 and 6,981 seconds. That is, GramAlign finishes in 32% and 44% of the time required by the next fastest algorithm.

MUSCLE in fast mode encountered a segmentation fault during the Root Alignment step while running on the longest test sequences, and so the execution time is not included in Figures 6 and 7.

#### Numerous Sequence Experiments

In order to compare the performance of MSA algorithms on data sets with many sequences, two sets of seven FASTA files each containing sequences with an average length of 100 residues were generated using Rose version 1.3. The first set of seven FASTA files contains protein sequences and the second set contains DNA sequences. In both sets, the first file contains 100 sequences, with each file increasing the number of sequences up to the seventh file, which contains 10,000 sequences.

**Figure 8:**
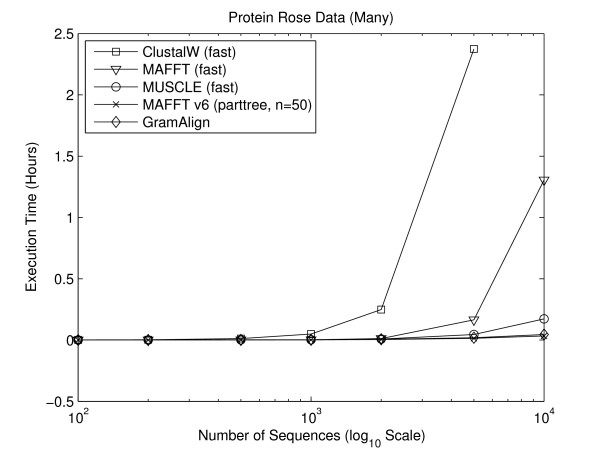
**Rose protein many**. Result of executing the fastest algorithms on the Rose-generated numerous protein sequence sets.

**Figure 9:**
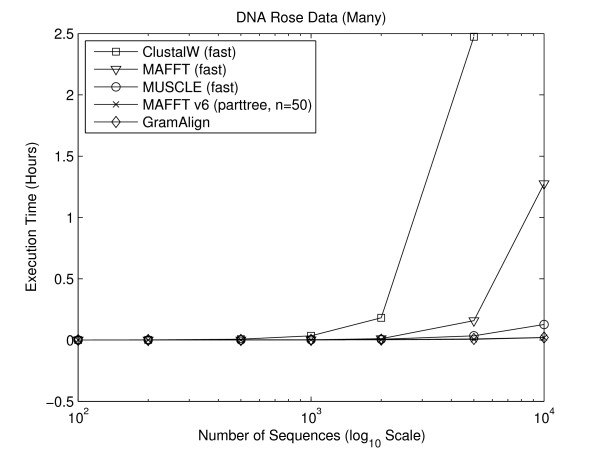
**Rose DNA many**. Result of executing the fastest algorithms on the Rose-generated numerous DNA sequence sets.

As shown in [30], the authors of MAFFT added a new heuristic method for generating a spanning tree referred to as "PartTree". The increase in performance is dramatic and intended for data sets involving many sequences. Thus, for this set of experiments, MAFFT version 6.240 was added with the command line mafft --retree 1 --parttree --partsize 50, which matches the fastest algorithm presented in [30]. Figures 8 and 9 depict the execution time required for the fastest algorithms to align the seven large protein and DNA sequence sets, respectively. As the number of sequences increases, the difference in time required by GramAlign and MAFFT v6 compared to the other algorithms also increases. In particular, on the sets containing 10,000 protein and DNA sequences GramAlign completes the alignments in 162 and 68 seconds and MAFFT v6 completes the alignments in 119 and 71 seconds, while the next closest algorithm, MUSCLE in fast-mode, requires 621 and 456 seconds. That is, GramAlign finishes in 26% and 15% of the time required by the next fastest algorithm other than MAFFT v6.

The results imply the promising viability of the proposed algorithm, especially when aligning either long or numerous sequences such as in whole-genome applications. Further, better alignment scores may be achieved with little change in execution time via the user-alterable parameters.

## Conclusion

The primary goal of this work was to introduce a computationally-efficient progressive alignment algorithm which can be used for aligning large data sets. The grammar-based distance work presented in [20] was adapted to generate an estimation of the proper order in which sequences are to be aligned. Additionally, a merged amino acid alphabet was determined to allow an improved grammar-based distance when operating on protein sequences. Results from extensive alignments were presented in an attempt to study the overall quality of the resultant alignments as well as the computation time necessary to achieve the alignments. Correctly aligning multiple biological sequences in an efficient amount of time is an important and challenging problem with a wide spectrum of applications. In this work, we adapt existing ideas in a novel way introducing innovative improvements. The proposed algorithm achieves reasonable alignments compared to existing methods while significantly reducing execution time. Future work will focus on determining the best set of user-defined parameters for generating the highest overall SP and TC scores.

## Availability

The current version of GramAlign may be run on-line, or the source code may be downloaded from the web server .

## Authors' contributions

DJR thought of applying the natural transitivity of the LZ grammars to the recursive division of the distance matrix, implemented the entire algorithm, performed all evaluations and drafted the initial manuscript. HHO conceived the idea of using an LZ grammar for progressive alignment. KS collaborated with HHO and DJR in the development of the algorithm and preparing the final manuscript. All authors read and approved the final manuscript.

## Supplementary Material

Additional file 1 An archive of the source code for the version of GramAlign at the time of publishing. An executable may be generated by unzipping this file and using an ANSI C compiler to build the code. Click here for file
